# Characterization of LGR5 stem cells in colorectal adenomas and carcinomas

**DOI:** 10.1038/srep08654

**Published:** 2015-03-02

**Authors:** Ann-Marie Baker, Trevor A. Graham, George Elia, Nicholas A. Wright, Manuel Rodriguez-Justo

**Affiliations:** 1 Centre for Tumor Biology, Barts Cancer Institute, Barts and the London School of Medicine and Dentistry, Queen Mary University of London, London, UK, EC1M 6BQ; 2 Department of Histopathology, University College London, London, UK, WC1E 6BT

## Abstract

LGR5 is known to be a stem cell marker in the murine small intestine and colon, however the localization of LGR5 in human adenoma samples has not been examined in detail, and previous studies have been limited by the lack of specific antibodies. Here we used *in situ* hybridization to specifically examine *LGR5* mRNA expression in a panel of human adenoma and carcinoma samples (n = 66). We found that a small number of cells express *LGR5* at the base of normal colonic crypts. We then showed that conventional adenomas widely express high levels of *LGR5*, and there is no evidence of stereotypic cellular hierarchy. In contrast, serrated lesions display basal localization of *LGR5*, and the cellular hierarchy resembles that of a normal crypt. Moreover, ectopic crypts found in traditional serrated adenomas show basal *LGR5* mRNA, indicating that they replicate the stem cell organization of normal crypts with the development of a cellular hierarchy. These data imply differences in the stem cell dynamics between the serrated and conventional pathways of colorectal carcinogenesis. Furthermore we noted high *LGR5* expression in invading cells, with later development of a stem cell niche in adenocarcinomas of all stages.

L*GR5* (Leucine-rich repeat containing G protein-coupled receptor) is a gene encoding for a component of the Wnt receptor complex, which specifically acts as a receptor for a family of Wnt pathway agonists called R-spondins. In the mouse antrum, small intestine and stomach the *Lgr5*-positive stem cells *lineage-label* - after marking with a reporter, all differentiated cell lineages normally present in a crypt are seen to be clonally-derived from Lgr5+ cells^1^,^2^, and at least in mice, *Lgr5* has also been shown to lineage-label gastric and intestinal stem cells. LGR5 is likely to be a robust stem cell marker in the human intestine also: *in situ* hybridization shows localization of *LGR5* mRNA to the crypt base mirroring the architecture seen in the mouse^3^,^4^,^5^. We have shown that the clonal evolution of human intestinal stem cells is a neutral process, which closely resembles that seen in the murine crypt, and that human crypts house a similarly small number of functional stem cells^6^.

In colorectal adenomas our knowledge of stem cell dynamics is also heavily dependent on animal experiments. *Lgr5*+ cells have been shown to lineage-label within mouse small intestinal adenomas, and a considerable proportion of the crypt population expresses *Lgr5*, suggesting a population of hundreds of potential stem cells in each adenomatous gland^7^. But recent experiments using continuous clonal labeling in mouse models indicate that each crypt contains only a small number of functional or ‘working’ stem cells^8^: and hence it is likely that most of the *Lgr5*+ population are inconsequential for long-term maintenance of the stem cell pool^9^. These observations suggest that few stem cells actually contribute to tumor growth: if this were true for human colorectal adenomas, it would perhaps account for their observed low growth rate^10^.

In normal and adenomatous human colonic crypts, we see long-term lineage tracing from the crypt base using mitochondrial DNA mutations as clonal markers^6^, suggesting the stem cell population indeed resides in the crypt base. In human adenomas, there have been claims using aldehyde dehydrogenase 1^11^, crypt base cell markers such as MSH2, Bcl-2 and survivin^12^ and the putative cancer stem cell markers CD44, CD166 and EpCAM^13^ that progressive overpopulation with stem cells occurs during colon tumorigenesis and drives the development of colorectal carcinoma. However, none of these markers has been shown even in animal studies to effectively lineage-label colonic stem cells.

Studies of LGR5 in the human colon have been relatively few; Becker and colleagues used antibodies against LGR5 to show that in human adenomas LGR5-expressing cells were no longer restricted to the crypt base, but were found in ‘patches’ at the luminal surface^14^. A further study reported that the number of LGR5 immunoreactive cells with cytoplasmic localization was increased in adenomas, spreading from the gland base to the surface, again in a patchy distribution pattern^15^. Immunohistochemistry has also been used to show a negative relationship between LGR5 expression at the luminal surface of human adenocarcinomas and tumor stage^16^. However, the reliability of antibodies targeting LGR5 remains in doubt^17^,^18^. For this reason, several studies have utilized *LGR5* ISH to examine expression of *LGR5* in colorectal cancers^4^,^5^,^19^. It is of particular note that in regions of colorectal cancers displaying a glandular organization, expression of putative stem cell markers (such as *LGR5* and EphB2) have been reported to be upregulated at the base of the glandular structures, implying a stem-like population positioned at the gland base^5^. However, a detailed analysis of the *LGR5* stem cell architecture in the serrated pathway of colorectal tumorigenesis, and how it may differ from the classical adenoma/carcinoma progression has not been reported.

Here we use ISH to systematically study the localization of *LGR5* expressing cells in human hyperplastic polyps, adenomas of different histological sub-types (see Fig. 1) and adenocarcinomas of all stages, with the aim to detect alterations in their stem cell architecture.

## Results

### *LGR5* is expressed at the crypt base in normal colon and hyperplastic polyps

To determine if the expression of the stem cell marker *LGR5* is altered during human adenoma progression, we carried out chromogenic ISH on a panel of human FFPE hyperplastic polyps, adenomas and adenocarcinomas of all stages (n = 66).

We validated the ISH protocol by using a negative control probe targeting the bacterial gene *dapB* (Supplementary Fig. S1A). Furthermore we performed ISH with 3,3′-Diaminobenzidine (DAB)-based detection in addition to Fast Red-based detection, and verified that both detection methods identify *LGR5* expression within the same cells, thus there was no false positive signal that could be attributed to the method of detection (Supplementary Fig. S1B). As previously reported^20^, we found that in normal human colonic crypts, Ki67 (MKI67) expression was restricted to the lower quarter of the crypt, and cytokeratin-20 (KRT20) expression was only found on the luminal surface, with a portion of the crypt expressing neither proliferation (Ki67) or differentiation (KRT20) markers (Fig. 2A). Here we used chromogenic ISH to show that normal human crypts (n = 7) express *LGR5* only in a small number of cells at the very base of the crypt (Fig. 2A), consistent with reported isotopic ISH^3^,^10^ and expression patterns seen in murine colonic crypts^2^. We noted that normal small intestine also expresses *LGR5* mRNA in a similarly small number of cells at the crypt base (Supplementary Fig. S2A and B).

**Figure 1:**
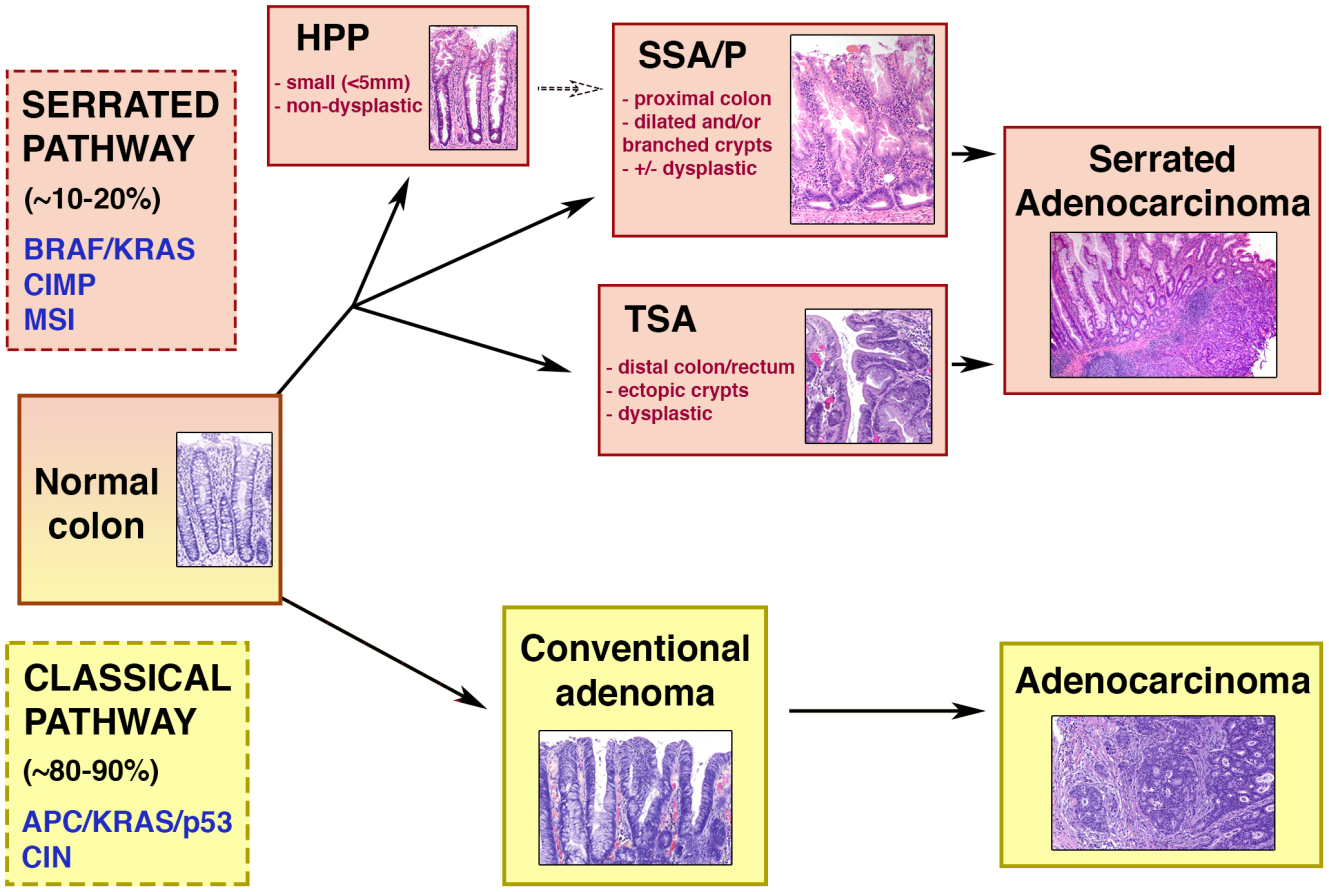
Schematic diagram of the relationship between histological states of the classical and serrated pathways. The upper half of the diagram represents the serrated pathway of colorectal carcinogenesis. Text in blue describes the genetic features of the serrated pathway (CIMP = CpG island methylator phenotype, MSI = microsatellite instability). The text in red describes some of the distinguishing features that are characteristic of the histological subtypes. The lower half of the diagram represents the classical pathway of carcinogenesis. Text in blue describes the genetic features of the classical pathway (CIN = chromosomal instability).

**Figure 2:**
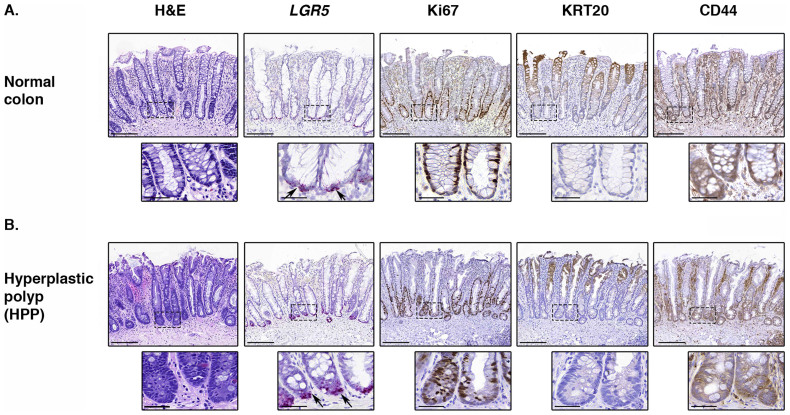
Stem cell architecture in human normal colon and hyperplastic polyps. Representative H&E staining, *in situ* hybridization (*LGR5*; pink) and immunohistochemical staining (Ki67, KRT20, CD44; brown) in human colon samples, illustrating the normal stem cell architecture (A, site = ascending colon) and the maintenance of a stem cell niche in hyperplastic polyps (HPPs, B, site = descending colon). Black arrows indicate LGR5 positivity at the base of crypts. Scale bars represent 200 micron, scale bars of inserts represent 50 micron.

To determine if the organization of the stem cell niche was disrupted in benign lesions, we examined the expression of *LGR5* in hyperplastic polyps (HPPs, n = 7). HPPs are defined as small, non-dysplastic serrated lesions of the colon, which are generally thought to exhibit little or no malignant potential, although certain subtypes may be precursors to sessile serrated adenomas/polyps^21^ (see Fig. 1). All of the HPPs described in this study were small lesions (<5 mm) and were validated independently by two expert pathologists as having no features suggestive of sessile serrated adenomas/polyps (SSA/Ps). As previously reported^20^, we found that all HPPs exhibited KRT20 expression that extends further down into the crypt than in normal crypts, and there was expansion of the Ki67+ population. Here we report that although *LGR5* remained confined to the crypt base, there was a significant increase in the intensity of *LGR5* expression in all hyperplastic crypts examined (Score of normal colon = 1.27 ± 0.12, HPP = 2.32 ± 0.23; p = 0.002 by the two-sided Student's t-test; Fig. 2B, Supplementary Fig. S3). Consistent with this observation, expression of the putative stem cell marker CD44 also appeared elevated compared to normal crypts, although remaining localized to the crypt base (Fig. 2B). We chose to use CD44 as an additional stem cell marker to validate the *LGR5* ISH, as it is one of the most widely used putative markers of stem cell populations in a broad range of normal and cancerous tissues^22^,^23^,^24^.

### *LGR5* is widely expressed throughout the glands of conventional adenomas

**Figure 3:**
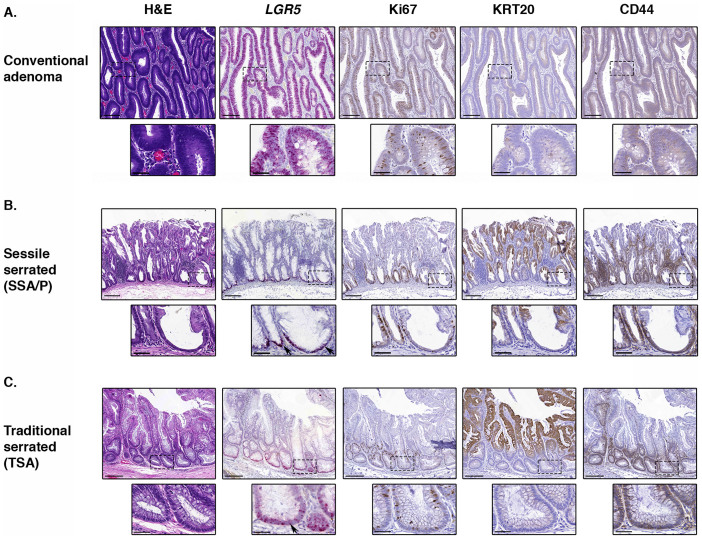
Stem cell architecture in human adenomas. Representative H&E staining, *in situ* hybridization (*LGR5*; pink) and immunohistochemical staining (Ki67, KRT20, CD44; brown) in human colon samples, illustrating the absence of stem cell architecture in a conventional adenoma (A, site = transverse colon), and the maintenance of a relatively normal stem cell niche in a sessile serrated adenoma/polyp (SSA/P, B, site = caecum) and a traditional serrated adenoma (TSA, C, site = rectum). Black arrows indicate LGR5 positivity at the base of crypts. Scale bars represent 200 micron, scale bars of inserts represent 50 micron.

**Figure 4:**
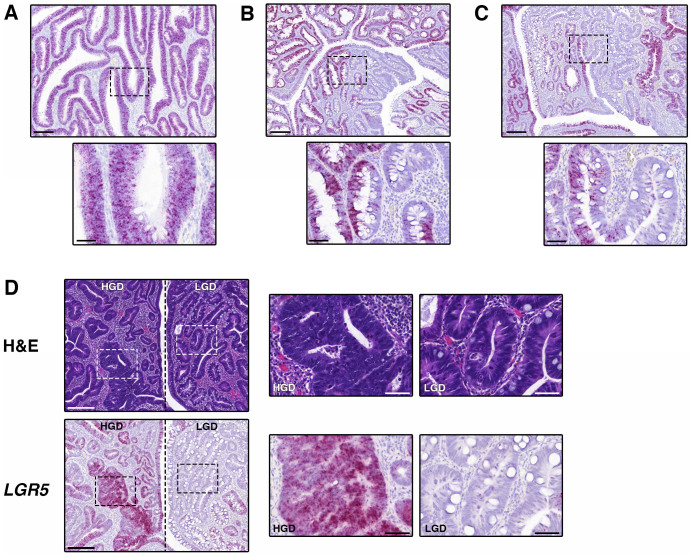
Diversity of *LGR5* expression in conventional adenomas. (A). Representative image of homogenous *LGR5* expression throughout the adenomatous glands (site = transverse colon). (B). Representative image of heterogeneous *LGR5* expression in a lesion (site = rectosigmoid). (C). Representative image of an adenomatous gland in which one branch expresses high *LGR5*, with the other half expressing low *LGR5*, suggestive of early dysregulation of the stem cell niche (site = rectosigmoid). (D). Representative images of *LGR5* expression within an adenoma (site = rectosigmoid) that contains regions of high-grade dysplasia (HGD) and low-grade dysplasia (LGD). Scale bars in A-D represent 200 micron, scale bars in inserts represent 50 micron.

We next examined conventional adenomas (n = 17). We report that within subregions of adenomatous tissue, *LGR5* is expressed in cells spread along the entire gland length and at a relatively uniform level (Fig. 3A, Fig. 4A), an expression pattern that has been previously documented^10^. Immunohistochemical analysis showed that the glands expressing high *LGR5* were often lacking KRT20 expression suggesting the cells were indeed in an undifferentiated state, and furthermore the expression of the stem cell marker CD44 was generally moderate throughout such adenomatous glands (Fig. 3A).

However our data suggests that such a uniformly high expression pattern is relatively uncommon in conventional adenomas. Within the majority of lesions we observed little uniformity of *LGR5* expression along the gland length and considerable variability in expression across the adenoma, though still with no apparent conservation of normal stem cell architecture (Fig. 4B). This ‘patchy’ expression of LGR5 has previously been reported in colonic adenomas in studies using LGR5-targeting antibodies^14^,^15^. Interestingly, we noted the presence of structures in which expansion of the *LGR5* compartment was restricted along the length of half of the adenomatous gland (Fig. 4C), suggestive of dysregulation of the stem cell niche. It is possible that these glands represent an early stage of the loss of stem cell architecture, and the stem cell population may be capable of further expansion until all cells in the gland express *LGR5*. We further noted that within regions of high-grade dysplasia (HGD), there was a generally higher level of *LGR5* expression than within regions of low-grade dysplasia (LGD; Fig. 4D). Additional examples of the heterogeneity of *LGR5* expression in conventional adenomas can be found in Supplementary Fig. S4.

### *LGR5* expression remains basal in serrated adenomas

Although the majority of colorectal cancers arise via the conventional adenoma-carcinoma pathway, approximately 10–20% of colorectal carcinomas are thought to arise from serrated lesions^25^. These tumors follow a different pathway of progression from adenoma to carcinoma (Fig. 1), and have a distinct genetic and epigenetic profile to their non-serrated counterparts^26^, and it has been proposed that serrated lesions can be identified by differential expression of certain markers^27^. We therefore sought to determine if the disruption of the stem cell niche was a feature of the crypts of sessile serrated adenomas/polyps (SSA/Ps) and traditional serrated adenomas (TSAs).

We found that SSA/Ps (n = 7) displayed expansion of the *LGR5*-expressing compartment, although unlike conventional adenomas we saw that *LGR5* in SSA/Ps remained generally localized to cells in the base of the crypt (Fig. 3B). The expression of *LGR5* was increased in SSA/Ps compared to normal crypts and HPPs (Score of SSA/Ps = 3.25 ± 0.13, HPP = 2.32 ± 0.23; p = 0.003 by the two-sided Student's t-test, Supplementary Fig. S3), consistent with a report suggesting that the putative stem cell marker CD133 is more highly expressed in SSA/Ps than HPPs^28^. The expression of CD44 was more extensive than normal or hyperplastic crypts, although still localized to the crypt base (Fig. 3B). SSA/Ps frequently displayed expansion of the Ki67+ population compared to normal crypts, and KRT20 expression was generally no longer confined to the upper third as it was in HPPs (Fig. 3B).

We also examined traditional serrated adenomas (TSAs, n = 6), and report that these also appeared to maintain a near-normal stem cell architecture. As with SSA/Ps, *LGR5* expression was significantly increased relative to normal crypts (Score of TSAs = 2.73 ± 0.28, normal colon = 1.27 ± 0.12, p < 0.0001 by the two-sided Student's t-test, Supplementary Fig. S3) yet restricted to the base of the crypts, and the expression patterns of Ki67, KRT20 and CD44 resembled that of the normal crypt (Fig. 2C).

### *LGR5* is expressed at the base of ectopic crypts in TSAs

**Figure 5:**
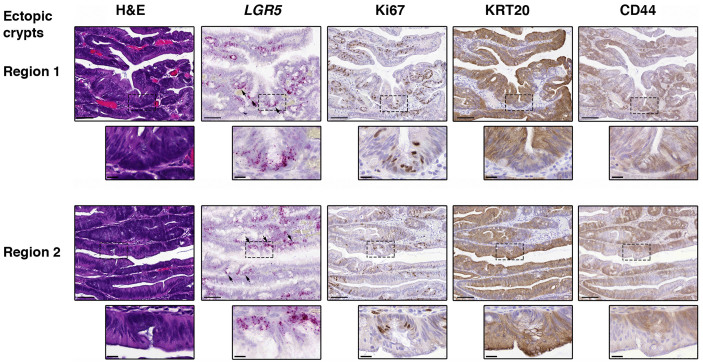
Expression of *LGR5* in ectopic crypts. Representative images showing expression of *LGR5* and Ki67 in ectopic crypts of a traditional serrated adenoma (TSA, site = sigmoid colon). Black arrows identify *LGR5* positivity at the base of ectopic crypts. Scale bars represent 100 micron, scale bars of inserts represent 20 micron.

Ectopic crypts are a defining feature of TSAs in which small crypts grow laterally from the side of the original crypt^20^. We identified regions containing ectopic crypts in the TSAs that were analyzed for *LGR5* expression, and present novel evidence to show that these crypts contain a small population of *LGR5*-expressing cells at the base. This suggests that the growth of ectopic crypts is supported by *LGR5*-positive cells within a ‘mini stem cell niche’ (Fig. 5). The ectopic crypts also displayed localization of Ki67, KRT20 and CD44 expression (Fig. 5) that closely resembled that of a normal crypt, suggesting that a cellular hierarchy is established within ectopic crypts.

### Carcinomas of all stages display basal localization of *LGR5*

**Figure 6:**
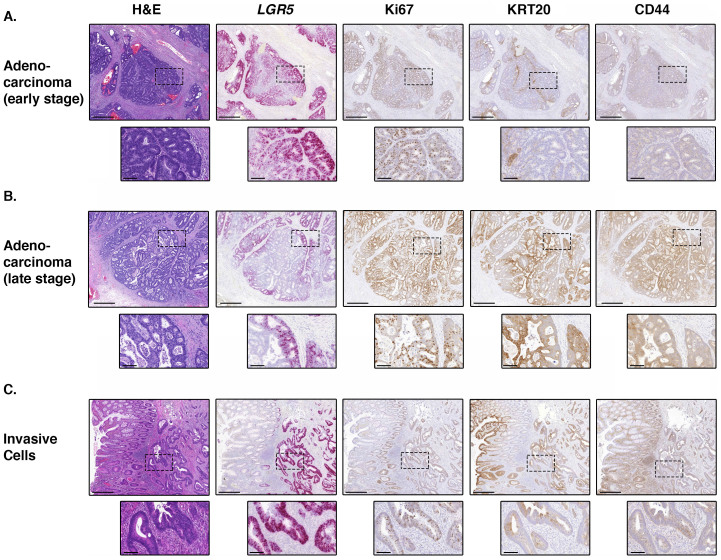
Expression of *LGR5* in invasion and adenocarcinoma. (A). Representative images showing basal *LGR5* expression in adenocarcinoma structures resembling crypts in an early stage cancer (pT1, site = sigmoid colon). (B). Representative images showing basal *LGR5* expression in adenocarcinoma structures resembling crypts in a late stage cancer (pT3, site = sigmoid colon). (C). Representative images showing universally high *LGR5* expression in an invasive cell population found within a conventional adenoma (site = sigmoid colon). Scale bars in A, B and C represent 500 micron, scale bars of inserts represent 100 micron.

It has been reported that human colorectal cancers can contain an *LGR5*-expressing stem cell niche at the base of tumor glands resembling crypts^4^,^5^. We therefore analyzed *LGR5* ISH and Ki67, KRT20 and CD44 IHC in 23 adenocarcinomas of all stages (pT1: n = 9, pT2: n = 3, pT3: n = 7, pT4: n = 4). Of these 23 adenocarcinoma samples, 18 were found to be suitable for *LGR5* ISH analysis, and consistent with the previous reports we generally observed *LGR5* expression in the proximal portion of the gland in adenocarcinomas of all clinical stages (Fig. 6A, B and Supplementary Fig. S5). The localization of Ki67 and KRT20 was also compartmentalized basally and therefore reminiscent of the normal stem cell architecture (Fig. 6A, B), suggesting a cellular hierarchy is indeed present. We did not note a difference in *LGR5* expression between stages, however we recognize that this analysis may be confounded by our small sample number, as well as by poor or inconsistent fixation of resection specimens, therefore we did not perform detailed quantitative analysis of *LGR5* expression in cancers.

We analyzed the level of Ki67, KRT20 and CD44 expression in the adenocarcinomas (n = 23) by using a four grade semi-quantitative scoring system with 0 representing no staining and 3 representing the strongest staining (Supplementary Fig. S6, A). We report that carcinomas of all clinical stages almost exclusively express moderate or high levels of Ki67 (Supplementary Fig. S6, B), even in invasive cells. This is in apparent contrast to a previous report^29^ in which low Ki67 was described at the invasive front, however such comparisons are confounded by inconsistency of staining protocols and inter-observer heterogeneity. The expression of KRT20 and CD44 displayed much more inter-sample heterogeneity with tumors displaying a large range of antigen expression (Supplementary Fig. S6, B), however our sample size was too small to permit a statistical comparison between clinical stages or sites of disease.

The expression of *LGR5* has been reported to be correlated with invasion and metastasis of gastric cancer^30^. We therefore sought to examine *LGR5* expression within invasive cell populations in our samples, and noted that *LGR5* expression was generally high in populations of invading cells (Fig. 6C), implying a massive expansion in stem cell number during this process. We noted that these small clusters of invasive cells generally express high CD44 and low KRT20 (Fig. 6C), indicating they are indeed in a non-differentiated state. In comparing the invasive front of early and late stage adenocarcinomas, we noted no clear difference between sample groups. However, we recognize that such analyses can be hampered by the confounding orientation of samples within FFPE blocks, and also by poor or inconsistent fixation of resection specimens.

## Discussion

LGR5 is accepted as the most reliable intestinal stem cell marker currently in use, however the expression and localization of the receptor in human adenomatous crypts or glands remains the subject of much debate. A previously published report has suggested that the organization of the cell hierarchy within murine adenomas closely resembles that of the normal crypt, with *Lgr5* expression remaining at the crypt base^7^. However there is also evidence to suggest that in the human colon, stem cell architecture is lost during the development of adenomas, and that expression of the colonic stem cell marker *LGR5* is widespread throughout adenomatous glands^10^. This report clarifies these discrepancies by providing new evidence that while conventional adenomas have a disrupted stem cell architecture as determined by frequent and widespread non-basal expression of *LGR5*, the stem cell architecture is remarkably conserved in HPPs, SSA/Ps and TSAs.

We detected significantly elevated levels of *LGR5* mRNA in all serrated lesions examined (HPPs, SSA/Ps and TSAs; n = 20), and this was generally accompanied by expansion of the proliferative (Ki67+) and differentiated (KRT20+) compartments. However *LGR5* expression remained universally basal and the stem cell architecture resembled that of a normal crypt, suggesting a stem cell niche was present and functional.

It is of particular interest that *LGR5* was expressed basally in the ectopic crypts of TSAs, with an associated cellular hierarchy. These structures extend laterally from crypts of a TSA, and little is known about the mechanism of their formation, although in the murine colon it has been proposed to be due to a repression of bone morphogenetic protein (BMP) signaling^31^. The observation that ectopic crypts express basal *LGR5* suggests that their growth is indeed sustained by cell division within a ‘mini stem cell niche’. Whether *LGR5*-positive stem cells migrate from the base of the adenomatous crypt to these ‘mini niches’, or a niche forms *de novo* due to changes in stromal signals (such as BMP) remains unclear.

This apparent conservation of the stem cell niche in serrated lesions is in striking contrast to the expression patterns in conventional adenomas. In all non-serrated lesions analyzed (n = 17), we saw that *LGR5* was no longer confined to the base of the glands and was generally widely expressed throughout the lesion, although considerable heterogeneity in the level of expression was often observed. We provided evidence of adenomatous glands that expressed *LGR5* only on one side or branch, a feature that implied during conventional adenoma progression *LGR5*-positive cells are no longer confined to the crypt base. This data is consistent with previous reports suggesting that mouse^7^ and human^10^ adenomatous glands display a dramatic increase in the number of *LGR5*-positive cells.

We note that in the murine colon, only a small percentage of the total *Lgr5*-expressing cells actually function as stem cells at any one time^8^, though they are all endowed with stem cell potential^9^. In addition, we have recently shown that normal human colonic crypts are sustained by a small number of ‘functional’ stem cells (similar to that of murine crypts), however adenomatous crypts display an elevated number of functional stem cells^6^. Thus, applying this insight to conventional adenomas, the large number of *LGR5*+ cells suggests a large pool of cells with stem cell potential, but not necessarily a large number of functioning stem cells.

LGR5 is reported to positively regulate invasion and metastasis of gastric cancer via a matrix metalloproteinase 2 (MMP2)-dependent mechanism^30^, however *in vitro* and mouse xenograft models of colorectal cancer have shown that LGR5 knockdown increases invasion^32^. Our analysis revealed that *LGR5*-positive cells are substantially enriched in invasive cell populations, suggesting that LGR5 can support invasion rather than inhibit it. These results imply that the plasticity of the stem-like state may be permissive for invasion, allowing the cells to disseminate from the glands of the adenoma and invade through the muscularis mucosae. This expansion in stem cell number that occurs upon invasion is also seen in lineage-labelled murine stem cells, whereby measurements of clone size and proliferative potential in invasive squamous cell carcinoma showed geometric expansion of the stem cell population^33^.

Interestingly, a previous report suggests that in established human colon carcinomas, *LGR5* expression remains basal and is exclusive from KRT20 expression^5^. We were able to examine *LGR5* expression in 18 cases of adenocarcinoma, and consistent with the findings of Merlos-Suarez and colleagues, we report that in the majority of established carcinomas with a glandular structure, compartmentalization of *LGR5* and KRT20 is present. Combined with the observation of universally high *LGR5* in invasive cells, this lends itself to the hypothesis that the *LGR5*-positive population is responsible for the early stages of invasion.

In summary, this study applied ISH to a panel of human FFPE normal colon, adenoma and carcinoma samples (n = 66) to show that conventional adenomas display extensive expression of the stem cell marker *LGR5*, and this expression is no longer restricted to the base of adenomatous crypts. However, in serrated lesions, although *LGR5* expression is upregulated, the basal localization is retained and the cellular organization within the crypt resembles the normal colon. These are significant observations, as there is little currently known about the histogenesis of serrated adenomas, although the origin of conventional adenomas has been intensively investigated^34^,^35^. Our findings may reflect an important difference in their origin and mode of progression. Moreover, we note that significant shortcomings in the routine diagnosis of serrated colorectal polyps have been reported^36^, and due to its consistent basal localization in serrated lesions, the examination of *LGR5* distribution might play a role in improving discrimination of these enigmatic lesions.

## Methods

All methods were carried out in accordance with approved institutional guidelines, and experimental protocols were approved by the Research Ethics Committee (07/Q1604/17 and 11/LO/1613). Samples were collected in accordance with UK Home Office regulations, with all patients giving informed consent.

### Patient samples

Formalin-fixed paraffin embedded (FFPE) human tissue samples (n = 66) were obtained from University College Hospital, London. Histopathological classification of each patient sample was independently carried out by two expert pathologists (NAW and MRJ).

### *In situ* hybridization (ISH)

ISH for *LGR5* expression was performed on 5 μm sections using the RNAscope 2.0 High Definition (Red, catalog number 310036, or Brown, catalog number 310035) assay according to the manufacturers instructions (Advanced Cell Diagnostics, Hayward, CA). Briefly, samples were baked at 60°C for 1 hour, followed by deparaffinization and incubation with Pretreat 1 buffer for 10 minutes at room temperature (RT). Slides were boiled in Pretreat 2 buffer for 15 minutes, followed by incubation with Pretreat 3 buffer for 30 minutes at 40°C. Slides were incubated with the relevant probes for 2 hours at 40°C, followed by successive incubations with Amp1 to 6 reagents. Staining was visualized with 3,3′-diaminobenzine (DAB) or Fast Red for 10 minutes, then lightly counterstained with Gill's haemotoxylin. RNAscope probes used were *LGR5* (NM_003667.2, region 560–1589, catalog number 311021), *POLR2A* (positive control probe, NM_000937.4, region 2514–3433, catalog number 310451) and *dapB* (negative control probe, EF191515, region 414–862, catalog number 310043). *LGR5* expression at the crypt base was quantified according to the five-grade scoring system recommended by the manufacturer (0 = No staining or less than 1 dot to every 10 cells (40× magnification), 1 = 1–3 dots/cell (visible at 20–40× magnification), 2 = 4–10 dots/cell, very few dot clusters (visible at 20–40× magnification), 3 = > 10 dots/cell, less than 10% positive cells have dot clusters (visible at 20× magnification), 4 = > 10 dots/cell. More than 10% positive cells have dot clusters (visible at 20× magnification)). At least 3 patients per sample group, with an average of 25 crypts scored per sample group were scored, a number that was limited by the number of well-orientated crypts that were present in each tissue section.

### Immunohistochemistry (IHC)

IHC was performed according to standard protocol. Briefly, 5 μm serial sections were dewaxed, rehydrated and immersed in 3% hydrogen peroxide for 20 minutes to quench endogenous peroxidase activity. Antigen retrieval was carried out at 95°C for 20 minutes in sodium citrate buffer (pH 6.0). After cooling, sections were incubated with blocking buffer (PBS supplemented with 2% goat serum and 1% bovine serum albumin) for 1 hour at RT. Primary antibodies were applied for 1 hour at RT (rabbit anti-human Ki67 [ab92742] at 1:2000 dilution, Abcam plc, Cambridge, UK; mouse anti-human CD44 [NCL-CD44-2] at 1:100, Novocastra Laboratories Ltd, Newcastle upon Tyne, UK; mouse anti-human cytokeratin-20 [M7019] at 1:75, Dako, Cambridgeshire, UK). Sections were then incubated with a biotinylated secondary antibody at RT for 45 min, followed by incubation with peroxidase-conjugated streptavidin solution at RT for 45 min. Visualization of antibody binding was carried out using DAB and sections were lightly counterstained using Gill's haematoxylin. Intensity of antigen expression in adenocarcinomas was semi-quantitatively scored using a four grade system, with 0 representing no expression, 1 representing weak expression, 2 representing moderate expression and 3 representing strong expression.

### Statistical analysis

Data are presented as mean ± standard error of the mean (SEM). Analysis was performed using the two-sided Student's t test, and considered statistically significant when the p value was less than 0.05.

## Author Contributions

The project was designed and conceived by N.A.W., experiments were performed by A.M.B., patient samples were selected by M.R.J., histopathological classification of samples was performed by N.A.W. and M.R.J., tissue processing was carried out by G.E., data were analyzed by A.M.B., T.A.G., N.A.W. and M.R.J. and the manuscript was written by A.M.B., assisted by T.A.G., N.A.W. and M.R.J.

## Supplementary Material

Supplementary Information Supplementary information
