# Supplementary material for: Characterization of LGR5 stem cells in colorectal adenomas and carcinomas
Source: Sci Rep. 2015 Mar 2;5:8654. doi: 10.1038/srep08654 (PMC4345329; doi:10.1038/srep08654)

**SUPPLEMENTARY TABLE, FIGURES AND LEGENDS**

**'Characterization of LGR5 stem cells in colorectal adenomas and carcinomas'**

Ann-Marie Baker, Trevor A. Graham, George Elia, Nicholas A. Wright, Manuel Rodriguez-Justo

**Supplementary Table 1**

Table showing the distribution of samples that were collected from the proximal colon, distal colon and rectum.

Proximal colon = caecum, ascending colon, hepatic flexure, transverse colon; Distal colon = splenic flexure, descending colon, sigmoid colon; Rectum = rectosigmoid, rectum.

HPP = hyperplastic polyp, SSA/P = sessile serrated adenoma/polyp, TSA = traditional serrated adenoma, CRC = colorectal cancer.

| Sample group | Proximal colon | Distal colon | Rectum | **Total** |
|---|---|---|---|---|
| Normal | 4 | 3 | 0 | **7** |
| HPP | 3 | 2 | 2 | **7** |
| SSA/P | 6 | 0 | 0 | **6** |
| TSA | 0 | 1 | 5 | **6** |
| Non-serrated adenoma | 5 | 8 | 4 | **17** |
| CRC | 7 | 11 | 5 | **23** |

**Supplementary Figure S1. Validation of ISH detection of *LGR5***

**A.** Representative images showing serial sections hybridized with the *LGR5* probe (left panel) or the *dapB* negative control probe (right panel). Scale bars represent 200 micron, scale bars of inserts represent 50 micron. Site = transverse colon.

**B.** Representative images showing serial sections hybridized with the *LGR5* probe, then detected with Fast Red (left panel) or DAB (right panel). It can be seen that *LGR5* is detected in the same cells, regardless of the detection method. Scale bars represent 100 micron, scale bars of inserts represent 20 micron. Site = sigmoid colon.

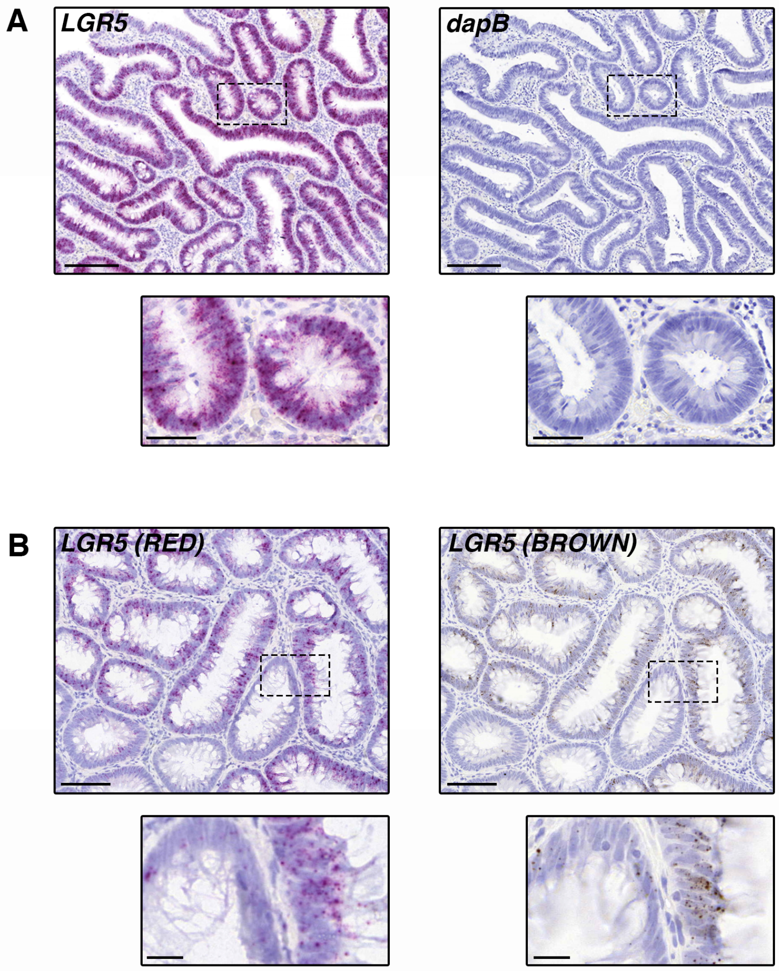

**Supplementary Figure S2. *LGR5* expression in small intestine**

**A.** Representative H&E staining, *in situ* hybridization (*LGR5*; pink) and immunohistochemical staining (Ki67, KRT20, CD44; brown) in a human small intestine sample. Scale bars represent 100 micron, scale bars of inserts represent 20 micron.

**B.** Graph showing average *LGR5* staining intensity at the base of normal colon and small intestine crypts. Mean number of crypts counted = 11 per group. Error bars represent mean ± SEM.

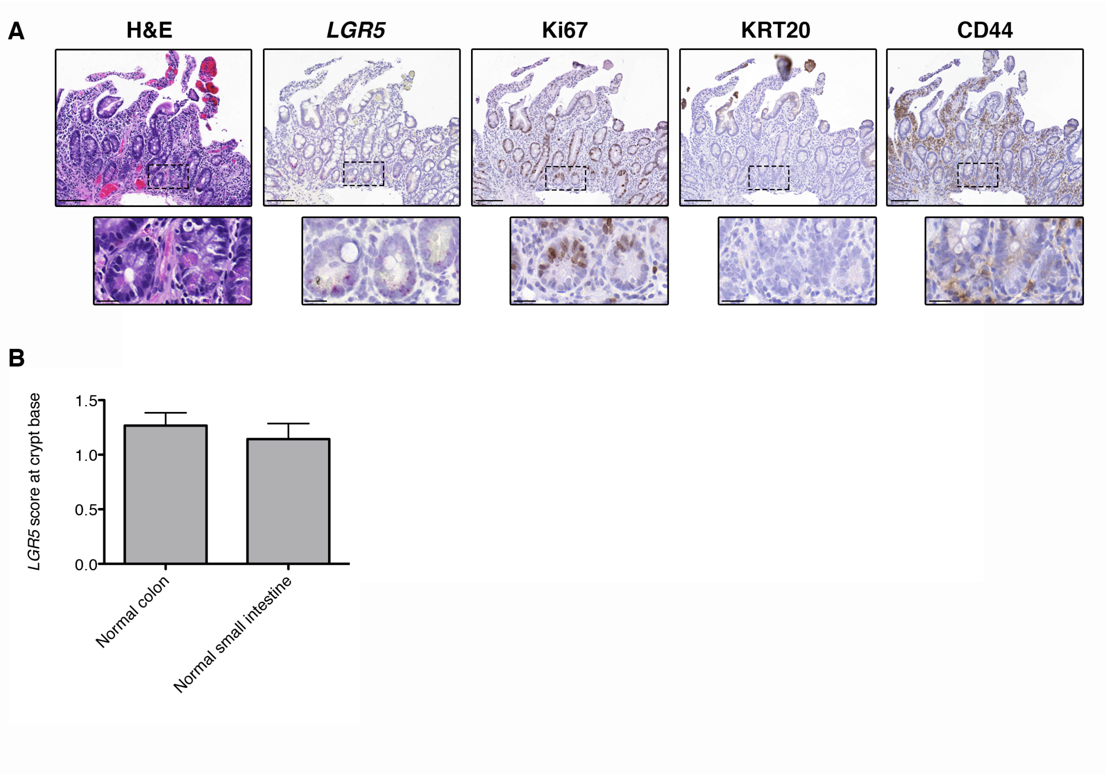

**Supplementary Figure S3. Quantification of *LGR5* at the crypt base**

Graph showing average *LGR5* staining intensity at the crypt base in normal colon, hyperplastic polyps (HPP), sessile serrated adenomas/polyps (SSA/P) and traditional serrated adenomas (TSA). Mean number of crypts counted = 23 per group. Error bars represent mean ± SEM, **p < 0.01 and ***p < 0.001 by the two-sided Student's t test.

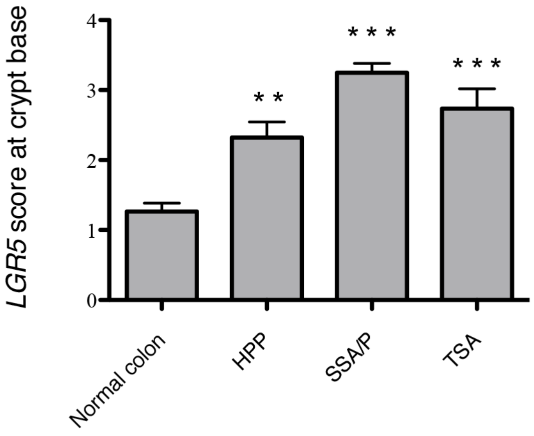

**Supplementary Figure S4. *LGR5* mRNA expression in non-serrated adenomas**

Representative H&E staining and *in situ* hybridization (*LGR5*; pink) in non-serrated adenomas from six patients. Scale bar represents 200 micron.

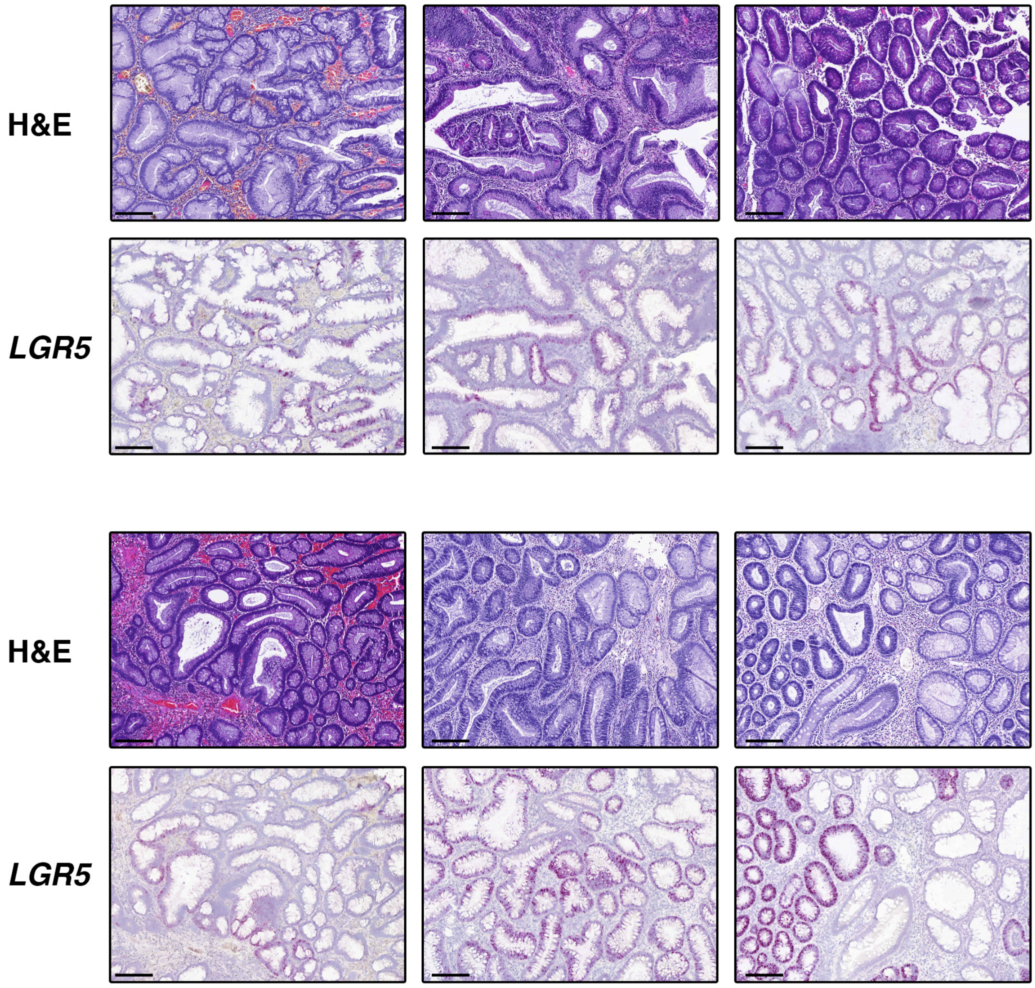

**Supplementary Figure S5. *LGR5* expression in adenocarcinomas**

Representative H&E staining and *in situ* hybridization (*LGR5*; pink) in adenocarcinomas of stages pT1 (**A.**), pT2 (**B.**), pT3 (**C.**) and pT4 (**D.**). Scale bars represent 200 micron.

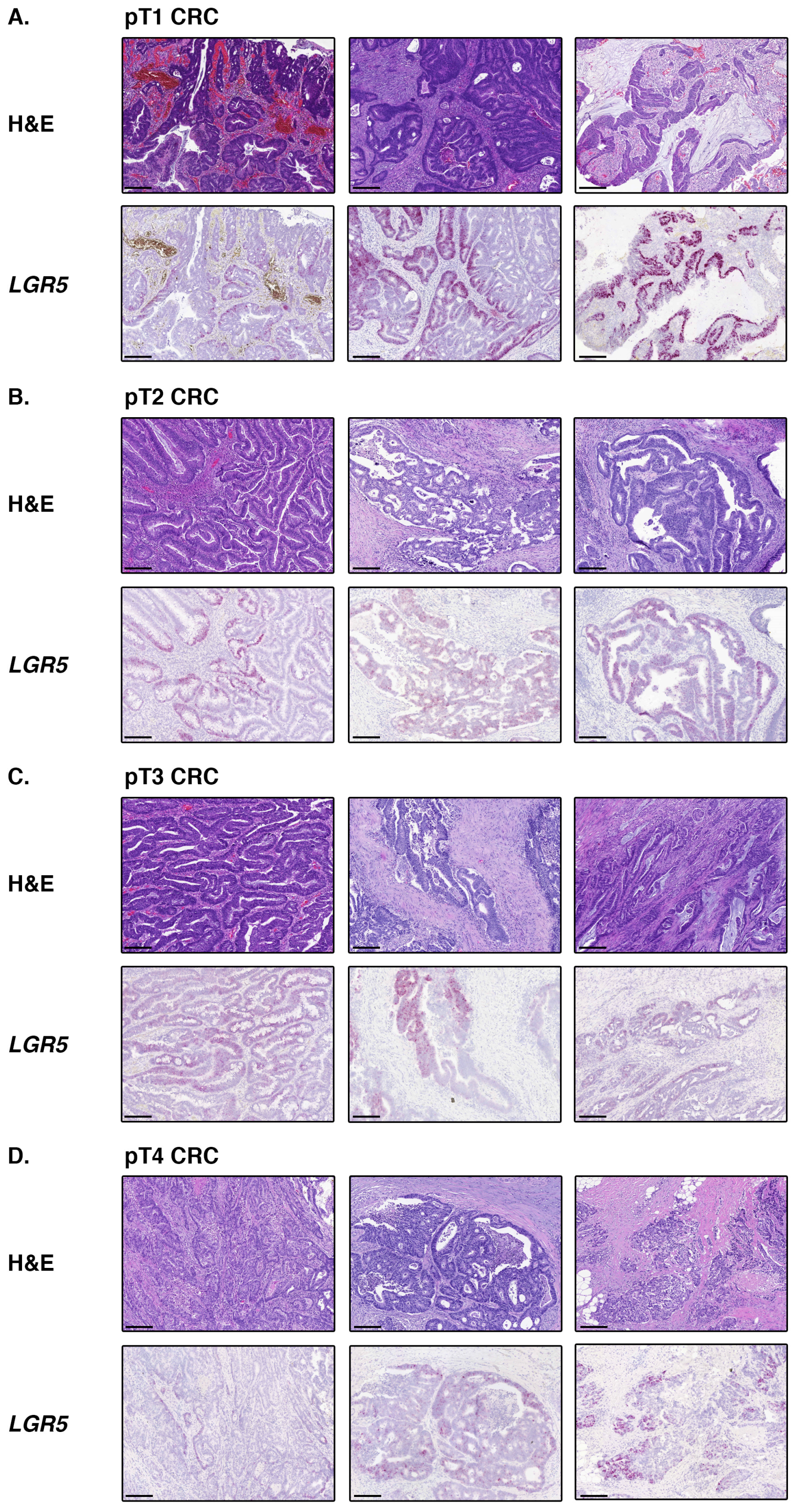

**Supplementary Figure S6. Semiquantitative analysis of Ki67, KRT20 and CD44 expression in adenocarcinomas**

**A.** Representative images showing no marker expression (0), weak expression (1), moderate expression (2) and strong expression (3). Scale bars represent 50 micron.

**B.** Graph showing the distribution of expression levels of Ki67, KRT20 and CD44 in adenocarcinomas (n = 23).

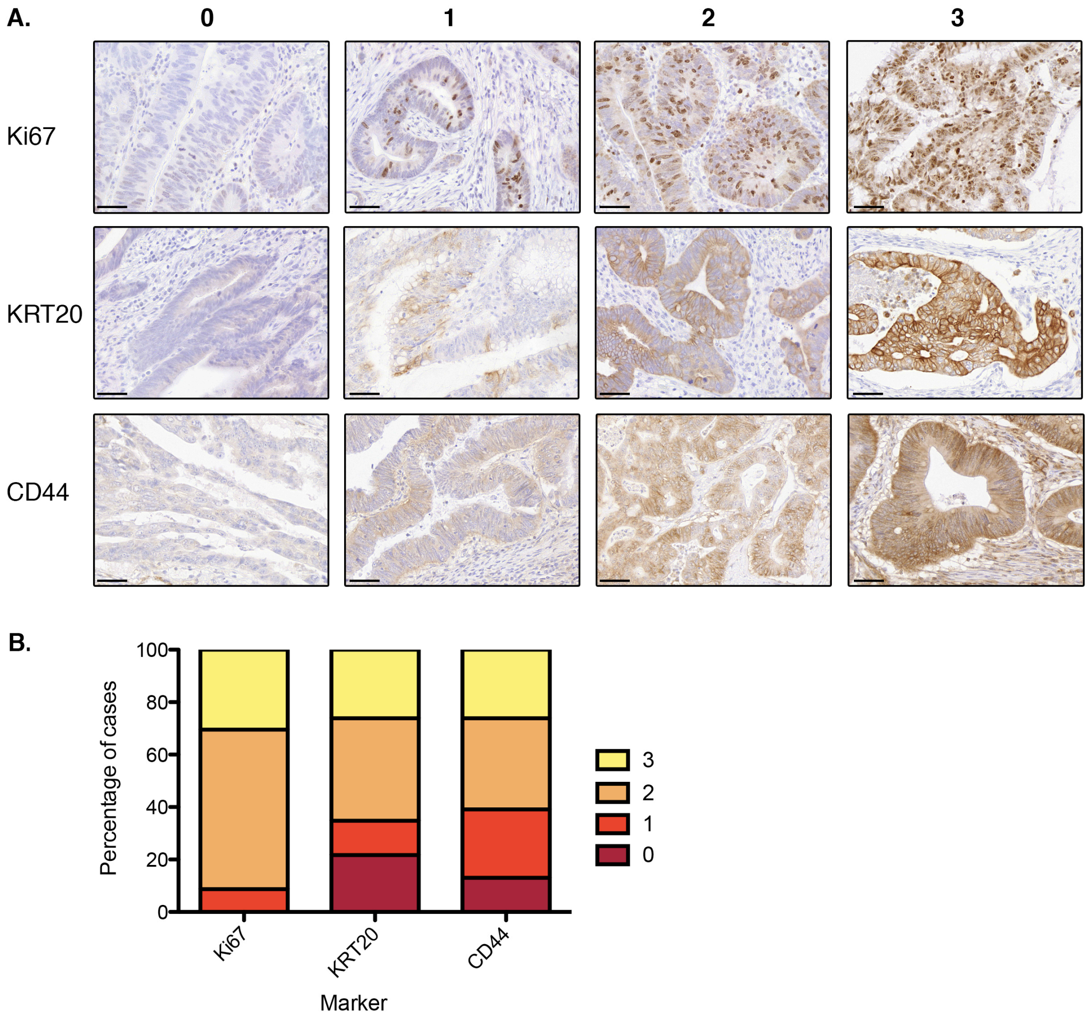

Supplement: Supplementary Information [file srep08654-s1.pdf]
